# Pigment epithelium-derived factor protects retinal ganglion cells

**DOI:** 10.1186/1471-2202-8-11

**Published:** 2007-01-29

**Authors:** Iok-Hou Pang, Hong Zeng, Debra L Fleenor, Abbot F Clark

**Affiliations:** 1 Alcon Research, Ltd., 6201 South Freeway, R3-24, Fort Worth, TX 76134, USA

## Abstract

**Background:**

Retinal ganglion cells (RGCs) are responsible for the transmission of visual signals to the brain. Progressive death of RGCs occurs in glaucoma and several other retinal diseases, which can lead to visual impairment and blindness. Pigment epithelium-derived factor (PEDF) is a potent antiangiogenic, neurotrophic and neuroprotective protein that can protect neurons from a variety of pathologic insults. We tested the effects of PEDF on the survival of cultured adult rat RGCs in the presence of glaucoma-like insults, including cytotoxicity induced by glutamate or withdrawal of trophic factors.

**Results:**

Cultured adult rat RGCs exposed to glutamate for 3 days showed signs of cytotoxicity and death. The toxic effect of glutamate was concentration-dependent (EC_50 _= 31 μM). In the presence of 100 μM glutamate, RGC number decreased to 55 ± 4% of control (mean ± SEM, n = 76; P < 0.001). The glutamate effect was completely eliminated by MK801, an NMDA receptor antagonist. Trophic factor withdrawal also caused a similar loss of RGCs (54 ± 4%, n = 60, P < 0.001). PEDF protected against both insults with EC_50 _values of 13.6 ng/mL (glutamate) and 3.4 ng/mL (trophic factor withdrawal), respectively. At 100 ng/mL, PEDF completely protected the cells from both insults. Inhibitors of the nuclear factor κB (NFκB) and extracellular signal-regulated kinases 1/2 (ERK1/2) significantly reduced the protective effects of PEDF.

**Conclusion:**

We demonstrated that PEDF potently and efficaciously protected adult rat RGCs from glutamate- and trophic factor withdrawal-mediated cytotoxicity, via the activation of the NFκB and ERK1/2 pathways. The neuroprotective effect of PEDF represents a novel approach for potential treatment of retinopathies, such as glaucoma.

## Background

Glaucoma, one of the world's leading causes of visual impairment and blindness [1], is characterized by excavation of the optic nerve head and selective apoptotic loss of retinal ganglion cells (RGCs), resulting in a progressive decline in visual function. Elevated intraocular pressure is a major risk factor for the development and progression of glaucoma, although the loss of vision in glaucoma patients does not always correlate with intraocular pressure and lowering pressure sometimes does not completely impede the disease process [2-4]. Clearly, ocular hypertension is not the exclusive cause of glaucomatous retinopathy, and additional mechanisms likely play a role in the degeneration of RGCs. In the past years, several additional mechanisms for glaucomatous optic neuropathy and retinopathy have been proposed, including disrupted retrograde transport of neurotrophic factors, glutamate toxicity, retinal and/or optic nerve ischemia, and immune abnormality [5-7]. These molecular events can eventually lead to apoptosis of RGCs. Unfortunately, the exact contribution of any of these factors in the pathogenesis of glaucomatous damage has not been unequivocally determined. It is probable that more than one etiology and multiple mechanisms are responsible in different patients and in different stages of glaucoma.

Despite our incomplete understanding of the disease processes and causes of RGC death, pharmacological protection of RGCs is under active investigation in ophthalmology research. Many neuroprotective strategies designed to prevent or delay the degeneration of RGCs are being evaluated, including some that are mechanism-specific. For example, glutamate receptor antagonists selectively protect against glutamate-induced cytotoxicity and may not have significant beneficial effects on other insults potentially involved in glaucoma. In contrast, other agents can protect RGCs against several toxic insults. These compounds, with their broader protective spectrum, are likely more useful as therapeutic agents for glaucoma. Pigment epithelium-derived factor (PEDF) appears to be one of these agents.

PEDF is a 50-kDa protein initially isolated from fetal human retinal pigment epithelial cells [8] and was later found to be expressed in various ocular tissues and cells, including the limbal region of the cornea and non-pigmented ciliary epithelial cells [9-12]. PEDF is also found in the brain and spinal cord, as well as non-neuronal tissues, such as endothelial cells and osteoblasts [13-15]. PEDF is a member of the serpin super-family of serine protease inhibitors [16]. However, unlike many serpins, PEDF does not inhibit serine proteases [17]. Instead, it exhibits potent antiangiogenic, neurotrophic and neuroprotective activities [13-15,18].

PEDF has broad neuroprotective effects in several neuronal cells and tissues. PEDF reduces glutamate-induced death of cerebellar granular cells, hippocampal neurons, and spinal cord motor neurons [19-21]. It decreases post-axotomy death of motor neurons and completely prevents atrophy of the surviving neurons [20]. In the retina, PEDF improves the survival of cultured mixed retinal cells under oxidative stress [22]. It also protects against light-induced damage to photoreceptor cells *in vivo *[23].

Intravitreal injection of recombinant PEDF or an adenoviral vector expressing PEDF was demonstrated to reduce retinal ischemia-induced RGC loss [24,25]. Although this animal model may not be relevant to glaucoma *per se*, the diverse protective effects of PEDF are nonetheless intriguing. We therefore tested PEDF in cultured adult rat RGCs to further characterize its potential protective effects against glaucoma-like insults.

## Results

**Figure 1:**
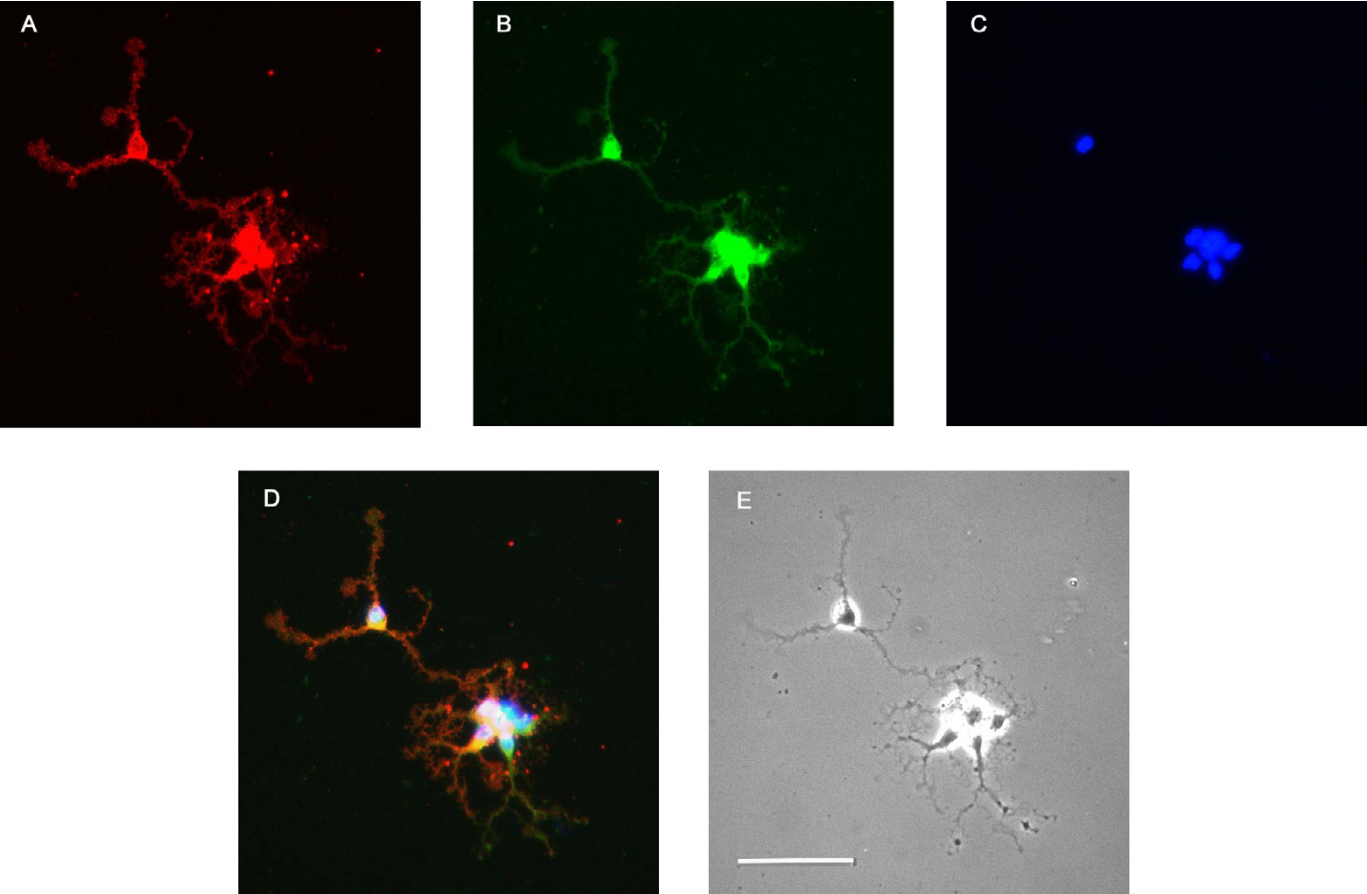
**Identification of cultured adult rat RGCs**. Cultured cells were co-labeled with anti-Thy-1 antibody (A), anti-NF-L antibody (B), and DAPI (C). (D) A digitally merged image simultaneously represents all three fluorescent labels. (E) The corresponding phase-contrast image. The cells were cultured for 3 days under control conditions. Scale bar = 50 μm.

The adult rat retinal cell cultures using the method described herein contained RGC-enriched retinal neurons. More than 90% of the cells were positively labeled with neuron-specific enolase antibody, indicating that the majority of cells are neurons (data not shown). Approximately 20–30% of these cells expressed Thy-1, and all Thy-1-positive cells were also positive for neurofilament-L (Figure 1). Both Thy-1 and neurofilament-L are selective cellular markers for RGC [26]. In contrast, cells in culture did not express marker proteins for other retinal cell types: they are negative for arrestin (photoreceptor), glial fibrillary acidic protein (GFAP; astroglia and Müller cell), glutamine synthetase (Müller cell), or ED1 (microglia). Less than 10% of the cells were labeled with the protein kinase Cα antibody (rod bipolar cells) (data not shown).

**Figure 2:**
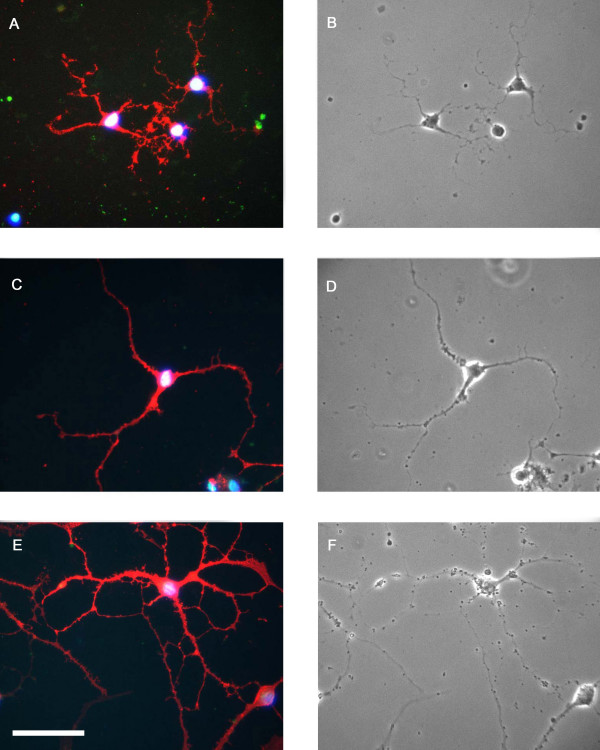
**Morphology of cultured adult rat RGCs**. The cells, cultured for 3 (A & B), 7 (C & D), and 11 (E & F) days, were labeled with anti-Thy-1 antibody (red) and DAPI nuclear stain (blue). (B), (D), are (F) are the corresponding phase-contrast images. Scale bar = 50 μm.

Morphologically, the Thy-1-positive cells had the characteristic appearance of neurons. After 2–3 days in culture, neurite outgrowth typically had 2–4 main branches of approximately 20–50 μm in length (Figure 2). As the culture period increased, the neurites lengthened and became more extensive, with most spanning greater than 100 μm in length after 7 or 11 days in culture (Figure 2).

**Figure 3:**
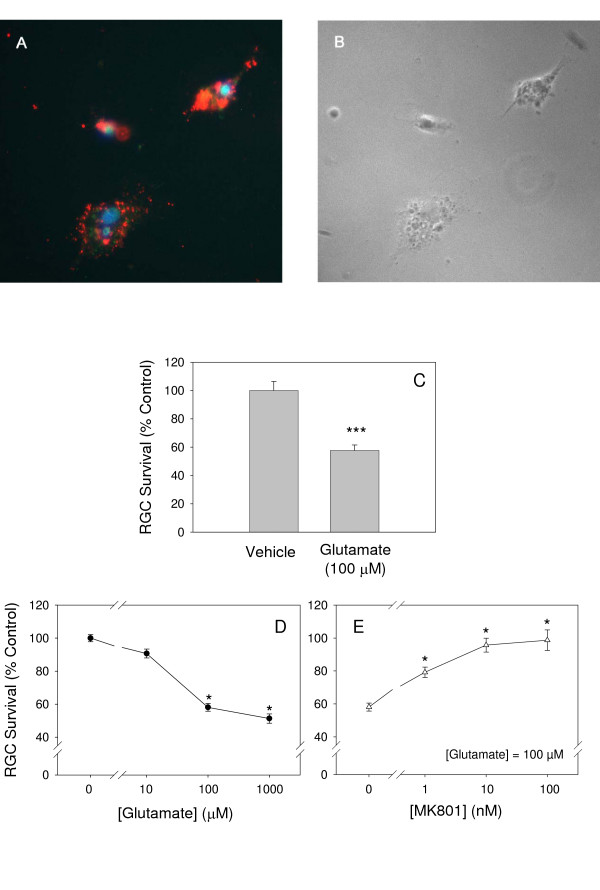
**Effect of glutamate on RGC morphology and survival**. Cells were treated with glutamate or vehicle for 3 days. (A and B) Morphology of RGCs with glutamate (100 μM) treatment. (A) The cells were labeled with anti-Thy-1 antibody (red) and DAPI nuclear stain (blue). (B) The corresponding phase-contrast image. (C) Effect of glutamate (100 μM) on RGC survival (n = 76). *** represents P < 0.001 vs. the control group by Student's t-test. (D) Concentration-dependent toxicity of glutamate on cultured RGCs (n = 6). * represents P < 0.05 vs. the control group by One-way ANOVA then Dunnett's test. (E) Effect of MK801 on glutamate-induced RGC toxicity. Cells were treated with the indicated concentrations of MK801 in the presence of 100 μM of glutamate (n = 6). * represents P < 0.05 vs. the glutamate alone group by One-way ANOVA then Dunnett's test. Cell survival in (C), (D), and (E) was assessed by manually counting the Thy-1-positive cells. Data are presented as mean ± SEM.

The RGCs appeared normal and healthy after 11 days in culture. For the sake of convenience, however our cell survival assays were performed using 3-day cultures. Under these conditions, there were 151 ± 7 Thy-1-positive cells/well (mean ± SEM, n = 124). Treatment with glutamate, an excitatory amino acid, damaged the cells. Glutamate (100 μM) caused significant changes in the morphology of many RGCs, including the loss of neurites, increased number of vacuoles in the cytoplasm, and compromised integrity of the plasma membrane (Figures 3A and 3B). In addition, there was formation of a greenish autofluorescence in the dying/dead RGCs, which, combined with the red fluorescent second antibody for Thy-1 detection, appeared yellow or orange (Figure 3B). Glutamate significantly (P < 0.001) reduced the number of RGCs in culture to 84 ± 6 cells/well (n = 76), corresponding to a 42% loss of RGCs (35–55% loss among studies) compared to control samples (Figure 3C). Glutamate toxicity was concentration-dependent with a calculated EC_50 _of 30.8 μM (Figure 3D). MK801, an antagonist for the N-methyl-D-aspartate (NMDA) glutamate receptor subtype, blocked the glutamate toxicity in a dose-dependent manner (Figure 3E). At 100 nM, MK801 completely prevented cell loss induced by 100 μM of glutamate. Agonists for non-NMDA glutamate receptors, such as kainate (100 μM, selective for ionotropic AMPA/kainate receptors), quisqualate (100 μM, activates both AMPA/kainate and metabotropic receptors), S-4-carboxy-3-hydroxyphenyl-glycine (50 μM, Group I metabotropic receptor agonist), L-carboxycyclopropylglycine (10 μM, Group II metabotropic receptor agonist), and L-(1)-2-amino-4-phosphonobutyrate (10 μM, Group III metabotropic receptor agonist) did not significantly affect RGC survival (data not shown). Glutamate toxicity appeared to be specific to the RGCs. At 100 μM, glutamate did not significantly affect Thy-1-negative cells in culture (data not shown).

**Figure 4:**
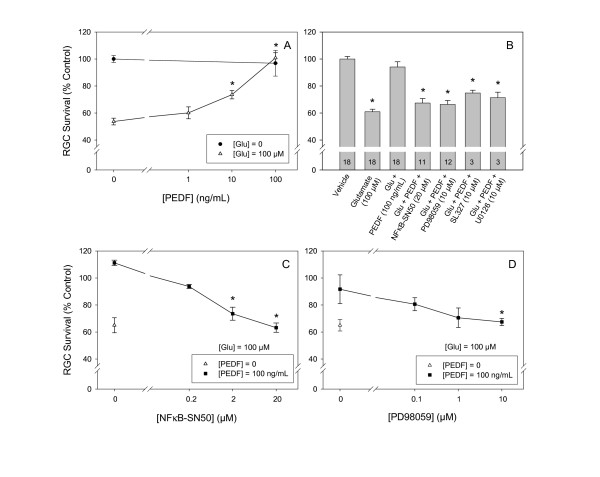
**Protective effect of PEDF against glutamate-induced RGC toxicity**. (A) Cells were treated with the indicated concentrations of PEDF in the presence or absence of 100 μM of glutamate (n = 6). (B) Effects of various compounds on PEDF protection against excitotoxicity. Numbers on the bars represent sample sizes. (C and D) Concentration-dependent reversal of the PEDF protection by NFκB inhibitor NFκB-SN50 and ERK1/2 inhibitor PD98059, respectively. * represents P < 0.05 vs. the respective control group by One-way ANOVA then Dunnett's test. Glu = glutamate.

Incubation of the RGCs with PEDF dose-dependently (EC_50 _= 13.6 ng/mL) protected against glutamate-induced toxicity. Complete protection was achieved by 100 ng/mL PEDF (Figure 4A). The same concentration of PEDF alone did not affect RGC survival or normal cell morphology. Although the PEDF receptor has not been well characterized, the biological effects of PEDF in other tissues and cell types are known to be mediated by the nuclear factor κB (NFκB) and/or extracellular signal-regulated kinases 1 and 2 (ERK1/2) cell signal transduction pathways [13-15]. Therefore, we evaluated the effects of inhibitors for these pathways on the protective action of PEDF in our RGC culture system. As shown in Figure 4B, the PEDF-mediated RGC protection against glutamate toxicity was abolished by either NFκB-SN50, a cell-permeable NFκB inhibitor peptide, or ERK1/2 inhibitors, PD98059, SL327 and U0126. The effects of NFκB-SN50 and PD98059 were concentration-dependent (Figures 4C and 4D). At the highest concentrations tested, these two compounds alone did not affect RGC survival (data not shown).

**Figure 5:**
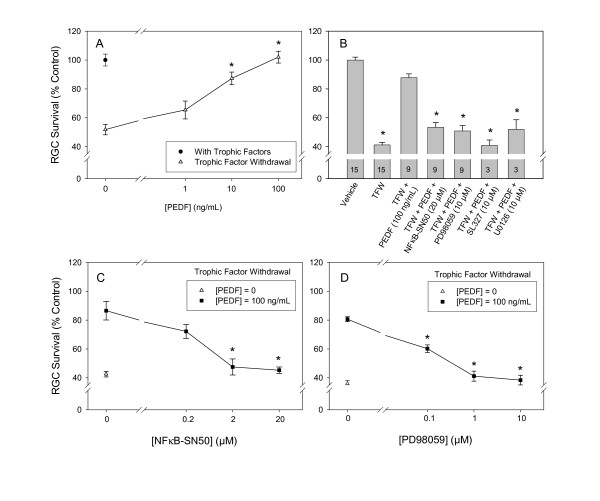
**Protective effect of PEDF against trophic factor withdrawal-induced RGC toxicity**. (A) Cells were treated with the indicated concentrations of PEDF in the presence or absence of trophic factors (n = 6). (B) Effects of various compounds on the PEDF protection against trophic factor withdrawal-induced cytotoxicity. Numbers on the bars represent sample sizes. TFW = trophic factor withdrawal. (C and D) Concentration-dependent reversal of the PEDF protection by NFκB inhibitor NFκB-SN50 and ERK1/2 inhibitor PD98059, respectively. Three trophic factors, bFGF, BDNF, and CNTF were removed from the culture medium in the trophic factor withdrawal group. * represents P < 0.05 vs. the respective control group by One-way ANOVA then Dunnett's test.

In addition to glutamate, the survival of RGCs in culture was also sensitive to trophic factor withdrawal. Removal of brain-derived neurotrophic factor (BDNF), basic fibroblast growth factor (bFGF), ciliary neurotrophic factor (CNTF), and forskolin from the culture media for three days caused a significant (P < 0.001) loss of RGCs, such that only 82 ± 6 cells/well (n = 60) remained, which corresponded to an average 47% RGC loss compared to controls. PEDF effectively protected the RGC against this insult in a concentration-dependent manner with a calculated EC_50 _of 3.4 ng/mL (Figure 5A). The PEDF protective effect on trophic factor withdrawal was also fully blocked by NFκB-SN50 and ERK1/2 inhibitors (Figures 5B, 5C, and 5D), similar to its effect against glutamate toxicity.

## Discussion

We have shown that glutamate was toxic to adult rat RGCs in culture, resulting in increased numbers of cells with aberrant morphology and decreased total numbers of surviving cells. This finding is similar to reports of glutamate toxicity in many other neurons. Glutamate is known to activate the NMDA, AMPA/kainite, and metabotropic glutamate receptors. Although RGCs express all of these receptor subtypes, the glutamate toxicity in our cultured RGC model is primarily mediated by NMDA receptors, because the NMDA receptor-specific antagonist MK801 blocked the response Agonists for the other glutamate receptors did not seem to be lethal to the cells. These results corroborate previous findings that the NMDA receptor is important for excitotoxicity in RGCs [27,28]. Nevertheless, because of controversies regarding glutamate-induced RGC toxicity, these data contradict other published findings. For example, some reported that both NMDA and kainite receptors contribute to RGC death (e.g., reference [29]), while others showed that only the kainite and not the NMDA receptor is responsible for RGC toxicity (e.g., reference [30]). Yet another group of investigators demonstrated that RGCs are resistant to glutamate or NMDA toxicity (e.g., reference [31]). The exact reasons for these disparities are not fully understood, but could be due to the different study conditions, which include the age and species of animals used, the presence or absence of other retinal cells in culture, or the duration and concentration of drug treatment. For example, in studies where excitotoxicity was not obvious, the incubation time was typically shorter (usually less than 24 h)[31] than that when excitotoxicity was observed (3 days in the current study)[27].

We also found that neurotrophic factors were essential for the survival of cultured RGCs. Numerous previous studies indicated that BDNF [32,33], CNTF [32,33], and bFGF [34] are essential survival factors. However, other trophic factors, such as nerve growth factor, glial cell line-derived neurotrophic factor, neurotrophins-3 and -4, have also been shown to be beneficial (reviewed in [35]). In the current cell culture, removal of BDNF, CNTF, and bFGF significantly reduced the survival of RGCs. Preliminary studies suggest that among these three trophic factors BDNF, in the presence of forskolin, was the most important for the health of the RGCs (unpublished observation).

Interestingly, not all RGCs were damaged by either type of insult in our culture system. Only about half of the RGCs were lost after either glutamate treatment or trophic factor withdrawal. Even with higher concentrations of glutamate or longer incubation times, we still did not observe a complete loss of the cells. Similar partial loss has been reported previously [27,29,36-40]. It is not clear if the neurons remaining after these insults differed biologically and functionally from those that perished. Dreyer and co-workers suggested that larger RGCs are more sensitive to glutamate toxicity [36]. However, we did not detect any size preferences of RGCs in susceptibility to either of the insults (unpublished observation).

PEDF protected cultured RGCs against both excitotoxicity and trophic factor withdrawal-induced cytotoxicity. Complete protection was observed at 100 ng/mL, a concentration shown to be neurotrophic and neuroprotective in other neurons [22,41,42]. This neuroprotective concentration of PEDF appears relevant because PEDF concentration in human vitreous and aqueous humor was determined to be approximately 0.5 to 3 μg/mL [43-49]. If RGCs in the retina are also exposed to similar levels of PEDF, PEDF may serve as one of the trophic factors that help sustain the health of RGCs. Interestingly, PEDF levels in aqueous humor of patients with advanced glaucoma are only about half of that of control eyes [48]. If PEDF levels in the vitreous and retina are also reduced in glaucomatous patients, the RGCs may be more susceptible to glaucomatous insults. The major sources of PEDF expression are in the retinal pigment epithelium, ciliary epithelium, and cornea [13-15]. In addition, RGCs themselves also make PEDF [10-12]. This locally produced PEDF may act as an autocrine effector and provide neurotrophic support for the RGCs.

Similar to other trophic factors, PEDF is expected to exert its biological effects by specifically binding and activating one or more receptors. However, PEDF receptors have not yet been fully characterized. Human retinoblastoma Y-79 cells, rat cerebellar granule neurons, cells in the ganglion cell layer, and inner segments of photoreceptor cells of bovine eyes have high affinity PEDF binding sites (K_d _= 3 nM) [50]. Most recently, a PEDF-binding protein, named PEDF-R, was identified from the pigment epithelium of human retina [51]. PEDF binds to this protein specifically and with high affinity. PEDF binding activates the protein and stimulates its phospholipase A_2 _enzymatic activity [51]. Currently, it is unclear whether PEDF-R is the only PEDF receptor subtype in the retina. Furthermore, its relationship to the cell signaling pathways responsible for the biological activity of PEDF is yet to be determined. Despite our incomplete knowledge of the PEDF receptor, the NFκB and ERK1/2 pathways appear to be involved in PEDF's activities [13-15]. Our studies with cultured RGCs also indicate that both pathways are essential for the neuroprotective effects of PEDF. Inhibition of either pathway was sufficient to abolish the protective effects of PEDF. Activation of the transcription factor NFκB induces expression of other neurotrophic factors, such as BDNF and nerve growth factor, as well as antiapoptotic genes, including Bcl2, Bcl-x, and superoxide dismutase [52,53]. ERK1/2 activation leads to modulation of other kinases, phosphatases, transcription factors, and regulators of apoptosis [54,55]. The identification of the down-stream effectors involved in the PEDF protection of RGCs awaits further studies.

The *in vitro *protective effects of PEDF suggest that PEDF may also protect RGCs *in vivo*. Indeed, intravitreal injection of PEDF significantly, albeit partially, protected against RGC loss and thinning of the inner retinal layers due to transient retinal ischemia in the rat [24]. Similar protection against the same insult and a reduction in apoptosis of the RGC were observed when adenoviral vector encoding PEDF was injected intravitreally [25]. Our results, together with these *in vivo *studies, suggest that PEDF is a potent and efficacious protectant for RGCs. PEDF should be a novel and effective therapeutic agent for glaucoma and other neuroretinopathies associated with RGC damage, provided obstacles to its delivery and metabolism can be overcome.

## Conclusion

Pigment epithelium-derived factor potently and efficaciously protects cultured adult rat RGC against glutamate- or trophic factor withdrawal-induced cytotoxicity. The protective effects of PEDF were mediated by activation of NFκB and ERK1/2 pathways. This neuroprotective effect of PEDF may lead to a novel approach for the treatment of retinopathies, such as glaucoma.

## Methods

### Retinal cell culture

Handling of animals in this study was conducted in accordance with the policies for use of animals in neuroscience research established by the Society for Neuroscience and by the Animal Care and Use Committee in Alcon. Isolation of retinal cells was modified from a previously reported procedure [27]. Briefly, adult Sprague-Dawley rats were euthanized by CO_2 _asphyxiation and their eyes enucleated. The retina from each eye was dissected and incubated in a papain solution, containing 2 mg/mL papain (Sigma, St. Louis, MO), 0.4 mg/mL DL-cysteine (Sigma), and 0.4 mg/mL bovine serum albumin (Sigma) in Neurobasal medium (Gibco/Invitrogen, Carlsbad, CA), for 25 min at 37°C, then washed 3 times with RGC culture medium [56] (Neurobasal/B27 medium containing 100 units/mL penicillin (Sigma), 100 μg/mL streptomycin (Sigma), 1 mM pyruvate (Gibco/Invitrogen), 2 mM glutamine (Gibco/Invitrogen), 5 μg/mL insulin (Sigma), 100 μg/mL transferrin (Sigma), 100 μg/mL bovine serum albumin (Sigma), 60 ng/mL progesterone (Sigma), 16 μg/mL putrescine (Sigma), 40 ng/mL sodium selenite (Sigma), 40 ng/mL thyroxine (Sigma), 40 ng/mL tri-iodothyronine (Sigma), 50 ng/mL BDNF (Biosource, Camarillo, CA), 10 ng/mL CNTF (Biosource), 10 ng/mL bFGF (Biosource), 5 μM forskolin (Sigma), and 1% fetal calf serum (Atlas Biologicals, Fort Collins, CO)). Retinal pieces were triturated by passing through a fire-polished disposable glass pipette until cells were dispersed. Cell density in the suspension was assessed with a Coulter counter (Beckman Coulter, Fullerton, CA). 1 × 10^6 ^to 3 × 10^6 ^cells/well were placed onto poly-D-lysine- and laminin-coated 8-well chambered culture slides (surface area = 0.69 cm^2^/well; Becton Dickinson, Franklin Lakes, NJ) and cultured in 95% air/5% CO_2 _at 37°C.

### Immunofluorescence

Cells were fixed with 3.7% formaldehyde in phosphate buffered saline (PBS)(EMD Chemicals, Gibbstown, NJ) at room temperature for 30 min, rinsed in PBS (Gibco) three times, and incubated for 1 h with PBS containing 0.02% saponin (Sigma) and the appropriate primary antibodies: Thy-1 (1:500; Chemicon, Temecula, CA), neurofilament-L (NF-L; 1:200; Serotec, Raleigh, NC), neuron-specific enolase (1:200; Chemicon), arrestin (1:200; Abcam, Cambridge, MA), protein kinase Cα (1:250; Oxford Biomedical Research, Oxford, MI), GFAP (1:500; Chemicon), glutamine synthase (1:500; Sigma), or ED1 (1:100; Santa Cruz Biotechnology). The primary antibody was then removed, cells rinsed again, and incubated for 30 min with fluorescence-labeled secondary antibodies (1:300), Alexa fluor 594-labeled goat anti-mouse IgG (Invitrogen/Molecular Probes, Carlsbad, CA), Oregon green-labeled goat anti-rabbit IgG (Invitrogen/Molecular Probes), or Alexa fluor 488-labeled rabbit anti-goat IgG (Invitrogen/Molecular Probes). The cells were subsequently incubated with DAPI solution (100 ng/mL; Sigma) for 5 min to stain their nuclei. The slides were rinsed with distilled water, covered with Fluoromount G (Southern Biotech, Birmingham, AL) and a cover glass (VWR, Westchester, PA). The retinal cells was examined by fluorescence microscopy and digital images obtained.

### Cytotoxic insults

For glutamate-induced toxicity studies, cells were pre-treated with vehicle or the indicated compounds for 30 min, followed by L-glutamate (0–1000 μM) (Sigma) for 3 days. For trophic factor withdrawal studies, three trophic factors, bFGF, BDNF, and CNTF, together with forskolin, were removed from the culture medium. Cells were cultured in this medium for 3 days.

### Quantification of cell survival

At the end of the incubation period, the cells were fixed then labeled for Thy-1 and DAPI. RGC survival was quantified by manually counting Thy-1-positive healthy cells in each well.

### Statistical analysis

Cell counts are expressed as mean ± SEM. Two-tailed Student's t-test was used to compare between two groups. One-way ANOVA followed by Dunnett's test was used to compare among three or more study groups. P < 0.05 is regarded as statistically significant.

## Authors' contributions

IHP contributed to the conception and design of the study, performed statistical analyses, and drafted the manuscript. HZ participated in the design of the study and carried out the cell assays. DLF participated in the design of the study, identified appropriate test agents, assisted in the interpretation of data, and helped to draft the manuscript. AFC participated in the conception and design of the study, assisted in the interpretation of data, and helped to draft the manuscript. All authors read and approved the final manuscript.
